# Mimicking chronic alcohol effects through a controlled and sustained ethanol release device

**DOI:** 10.1186/s13036-024-00428-1

**Published:** 2024-05-07

**Authors:** Wanil Kim, Jin-Ok Chu, Do-Yeon Kim, Soo-Hyeon Lee, Chang-Hyung Choi, Kyung-Ha Lee

**Affiliations:** 1 Department of Biochemistry and Institute of Medical Science, School of Medicine, Gyeongsang National University, Jinju, 52727 Republic of Korea; 2 Department of Cosmetic Science and Technology, Daegu Haany University, Gyeongsan, 38610 Republic of Korea; 3 Department of Pharmacology, School of Dentistry, Kyungpook National University, Daegu, 41940 Republic of Korea; 4 Department of Molecular Biology, Pusan National University, Busan, 46241 Republic of Korea; 5 School of Chemical Engineering, Yeungnam University, Gyeongsan, 38541 Republic of Korea; 6 Institute of Systems Biology, Pusan National University, Busan, 46241 Republic of Korea

**Keywords:** Ethanol, Ethanol-releasing device, Polydimethylsiloxane, Alcohol

## Abstract

Alcohol consumption, a pervasive societal issue, poses considerable health risks and socioeconomic consequences. Alcohol-induced hepatic disorders, such as fatty liver disease, alcoholic hepatitis, chronic hepatitis, liver fibrosis, and cirrhosis, underscore the need for comprehensive research. Existing challenges in mimicking chronic alcohol exposure in cellular systems, attributed to ethanol evaporation, necessitate innovative approaches. In this study, we developed a simple, reusable, and controllable device for examining the physiological reactions of hepatocytes to long-term alcohol exposure. Our approach involved a novel device designed to continuously release ethanol into the culture medium, maintaining a consistent ethanol concentration over several days. We evaluated device performance by examining gene expression patterns and cytokine secretion alterations during long-term exposure to ethanol. These patterns were correlated with those observed in patients with alcoholic hepatitis. Our results suggest that our ethanol-releasing device can be used as a valuable tool to study the mechanisms of chronic alcohol-mediated hepatic diseases at the cellular level. Our device offers a practical solution for studying chronic alcohol exposure, providing a reliable platform for cellular research. This innovative tool holds promise for advancing our understanding of the molecular processes involved in chronic alcohol-mediated hepatic diseases. Future research avenues should explore broader applications and potential implications for predicting and treating alcohol-related illnesses.

## Introduction

Alcohol misuse can lead to several negative physical and mental conditions. In 2021, approximately 60 million (21.5%) of American adults engaged in binge drinking [1]. Each year, alcohol-related deaths in the US total 95,000, with 68,000 occurring in males and 27,000 in females [2] . Consequently, alcohol consumption has a negative impact on society and the economy, and both acute and long-term alcoholism can cause major health issues. According to the International Classification of Diseases, Tenth Revision, alcohol misuse contributes to more than 25 diseases, such as diabetes, cancer, neuropsychiatric disorders, and cardiovascular diseases [3].

The liver performs vital functions in the body, but liver ailments, also known as hepatic disorders, can pose serious harm to the body. Hepatic diseases can originate from various factors, including parasitic and viral infections, hereditary genetic defects, and liver toxins, such as alcohol [4, 5]. Liver diseases are prevalent across the globe and are quite prevalent in Asia [6]. The liver, being the primary location of alcohol metabolism, becomes the target of alcohol-mediated harm, resulting in conditions such as fatty liver disease, alcoholic hepatitis, chronic hepatitis, liver fibrosis, and cirrhosis [7]. Even with minimal alcohol consumption, patients with hepatitis B and C face a higher risk of developing cancer than non-drinkers [8]. The causes and signaling pathways of alcohol-induced liver disorders have been extensively studied at clinical, animal, cellular, and molecular levels. Genetic predispositions linked to PNPLA3, ALDH2, CYP2E1, HSD17B13, FAF2, and NFE2L2 have been associated with alcoholic liver illnesses [9–13]. However, predicting and treating liver disorders resulting from prolonged alcohol consumption remains challenging.

Direct alcohol treatment of cell culture medium is frequently used to simulate acute alcohol exposure in organisms for a comprehensive understanding of the cellular and molecular mechanisms of acute alcohol-mediated liver damage [14]. However, mimicking chronic alcohol exposure at the cellular level is challenging due to ethanol evaporation from the culture medium, leading to potential misinterpretation of long-term cellular reactions [15]. Numerous techniques have been used to overcome this hurdle, including sealing the culture plate to reduce evaporation, adding ethanol regularly to the culture media, saturating the incubator’s air with ethanol, and using alcohol-releasing devices [16–18]. The most frequently employed method involves providing ethanol by changing the medium every 24 hours [18]. However, this approach results in fluctuating alcohol concentrations throughout cultivation, potentially causing medium contamination, thereby compromising the study’s validity and leading to results misinterpretation [17]. An alternative method to maintain cell exposure to ethanol is to saturate the chamber atmosphere above the culture media with ethanol or seal the culture dishes with Parafilm® [15]. However, during extended (>24 h) cultivation, these techniques may interfere with the normal exchange of oxygen and carbon dioxide, compromising cell viability. Therefore, developing a straightforward and suitable method for examining the physiological reactions of hepatocytes to long-term alcohol exposure becomes crucial. Additionally, the development of a method for the sustained and regulated release of ethanol over an extended period, such as several days, is imperative.

Previously, we developed a small, floating glass capillary system to release ethanol over several days [17]. The ethanol concentration in the culture medium was controlled by changing the number of capillaries. However, the capillary’s size limited its use to dishes larger than 35 mm, necessitating replacement to maintain the ethanol concentration beyond three days.

In this study, we developed a simple, reusable, and controllable device to overcome ethanol evaporation during continuous exposure. The ethanol concentration in the culture medium was tested to determine whether it remained consistent over several days by installing a simple carrier for continuous ethanol release into the culture dish.

## Results

### Fabrication of ethanol-release device

**Fig. 1:**
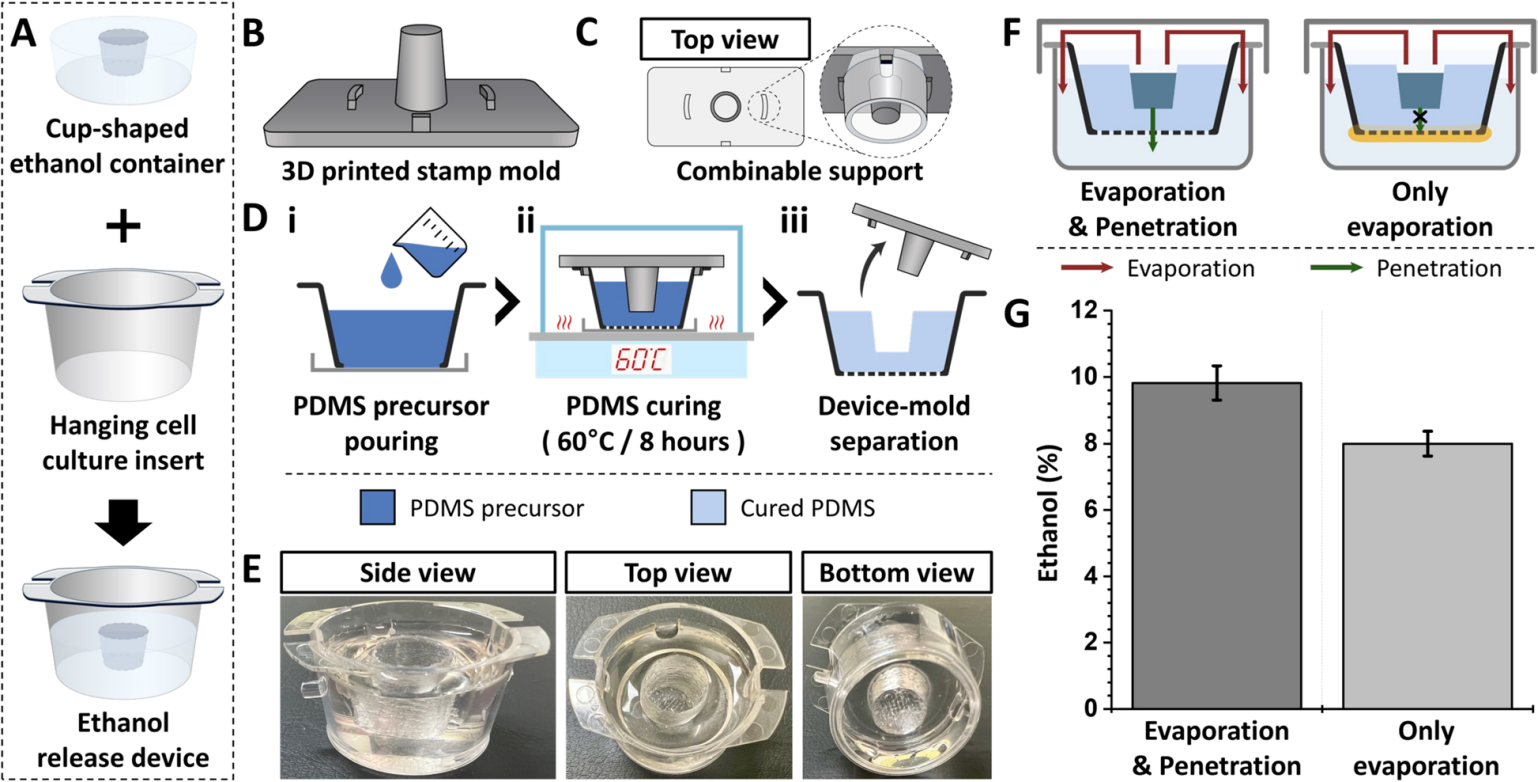
Fabrication of ethanol-release device. **A** Schematic showing ethanol-release device fabricated by the assembly of PDMS ethanol container and commercially available culture insert. Schematics showing (**B**) 3D view and (**C**) top view of the 3D printed mold. **D** Schematics showing the sequential procedure for creating an ethanol-release device. **E** Photographs showing a three-dimensional view of the prototype of the ethanol-release device. **F** Schematic showing the release pathway of ethanol. **G** Plot showing the proportion of release pathways for ethanol

Ethanol, with its high vapor pressure, poses challenges for simulating ethanol exposure in biological systems over time due to easy evaporation. To overcome these limitations, we designed a novel device that can continuously regulate ethanol release. The fabrication process for this ethanol-release device is illustrated in Fig. 1. The ethanol-release device consisted of a cup-shaped ethanol container and a commercially available hanging cell culture insert, as shown in Fig. 1A. To prepare the cup-shaped ethanol container, we created a 3D-printed polylactic acid (PLA) mold (Fig. 1B). The mold featured a cylindrical structure in the center with surrounding support structures, facilitating proper alignment of the components during the molding process (Fig. 1C).

The prepared mold was secured onto the cell culture insert containing the polydimethylsiloxane (PDMS) precursor that was prepared by mixing the PDMS prepolymer and curing agent (10:1 ratio), ensuring that the central cylindrical part was fully submerged (Fig. 1D-i). The bottom surface of the culture insert was sealed to prevent leakage. Next, the PDMS precursor was cured in an oven set at 60 °C for 8 h (Fig. 1D-ii). Finally, we completed the ethanol-release device by separating the mold from the cured PDMS (Fig. 1D-iii). The three-dimensional view of the fabricated ethanol-release device is shown in Fig. 1E.

The ethanol-release device was designed to release ethanol through two distinct routes. While one route involved evaporation in the upward direction, the other involved penetration through the bottom PDMS container (Fig. 1F). To achieve a stable and sustained ethanol release, the microwells should be saturated with ethanol through evaporation, allowing simultaneous dissolution in water. Additionally, ethanol can also be released through the bottom of the PDMS container. To validate this assumption, we compared our device with a configuration in which the bottom surface was sealed with a glass slide. Ethanol (0.5 mL) was introduced into the storage space of the container, and the device was inserted into a microwell plate containing water. The quantity of ethanol in the aqueous solution was measured 24 h after the concentration reached a steady state. The amount of ethanol from each microwell was determined using UV-vis spectroscopy (λ_abs_~962 nm). The plot shown in Fig. 1G exhibits that approximately 80% of the ethanol release occurred through evaporation, with only 20% penetrating through the bottom of the PDMS container. A small error bar indicates that this device enables consistent and sustained release of ethanol (Fig. 1G).

### Reusable and adjustable ethanol-releasing devices for the biological system

**Fig. 2:**
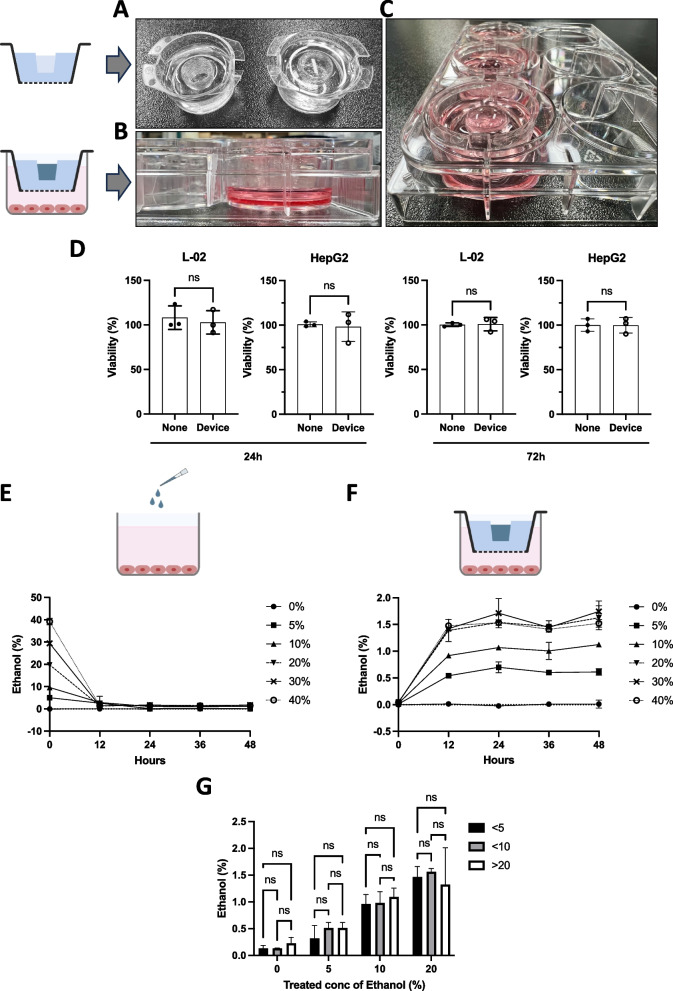
Reusable and adjustable ethanol-release device. **a** Top view of the PBS-containing ethanol-releasing device. **b** Side view of PBS-containing devices placed in a culture medium with 6-well plates. **c** Top view of PBS-containing devices placed in a culture medium with 6-well plates. **d** Cytotoxicity of PBS-containing PDMS devices in L-O2 or HepG2 cells. The viability of devices not positioned well was set to 100. **e** Ethanol was directly added to the cell culture medium of L-02 cells, and the ethanol concentration in the culture medium was measured at indicated time points. **f** Ethanol-containing PDMS devices were placed in the 6-well plates, and the ethanol concentration in the culture medium of L-02 cells was measured at indicated time points. **g** PDMS devices used less than five times, ten times, or 20 times were exposed to indicated ethanol concentrations, and the ethanol concentration in the culture medium of L-02 cells was measured

The biocompatibility and functionality that controlled the sustained release of ethanol from the PDMS devices were crucial for their application in cellular systems. The PDMS devices were filled with phosphate buffered saline (PBS) in the middle space (Fig. 2A) and placed in a 6-well plate containing the culture medium (Fig. 2B and C). To determine the biocompatibility of PDMS devices, we tested their cytotoxicity. We positioned the PDMS devices filled with PBS in culture media containing L-02 or HepG2 cells, which are frequently used in liver research. No cytotoxicity was observed after 24 h or 72 h of incubation with the PDMS device (Fig. 2D), suggesting that the PDMS device system can be used in biocompatible applications.

Next, we compared the ethanol concentration in the culture medium under various conditions, including the direct addition of ethanol to the medium and the placement of ethanol-containing PDMS devices on the culture plate. When ethanol was introduced directly into the medium, its concentration declined rapidly and reached near-zero levels after 24 h, independent of the initial ethanol concentration (Fig. 2E). In contrast, when PDMS devices were used, the ethanol concentration in the medium gradually increased over 12 h and remained sustained, correlating with the ethanol concentration in the PDMS devices (Fig. 2D). Increasing ethanol concentrations (0, 5, 10, and 20%) in the PDMS devices led to a corresponding concentration-dependent increase in the culture medium. However, concentrations exceeding 20% in the PDMS devices did not increase the ethanol concentration in the culture medium.

To assess the reusability of PDMS and minimize experiment costs and time, we added various ethanol concentrations to the PDMS devices with usage cycles of less than five times, less than 10 times, or more than 20 times. After a 24-h incubation, the ethanol concentration in the culture medium was measured. For reuse, the PDMS devices were first washed with 70% ethanol and subsequently rinsed with PBS. Sterilization was then achieved through UV irradiation. The number of reuse cycles did not affect the ethanol-release properties of the PDMS devices (Fig. 2G). These results suggest that PDMS devices can effectively control ethanol concentration in the culture medium, and biocompatible PDMS devices are reusable.

### Long-term exposure to ethanol using PDMS devices

**Fig. 3:**
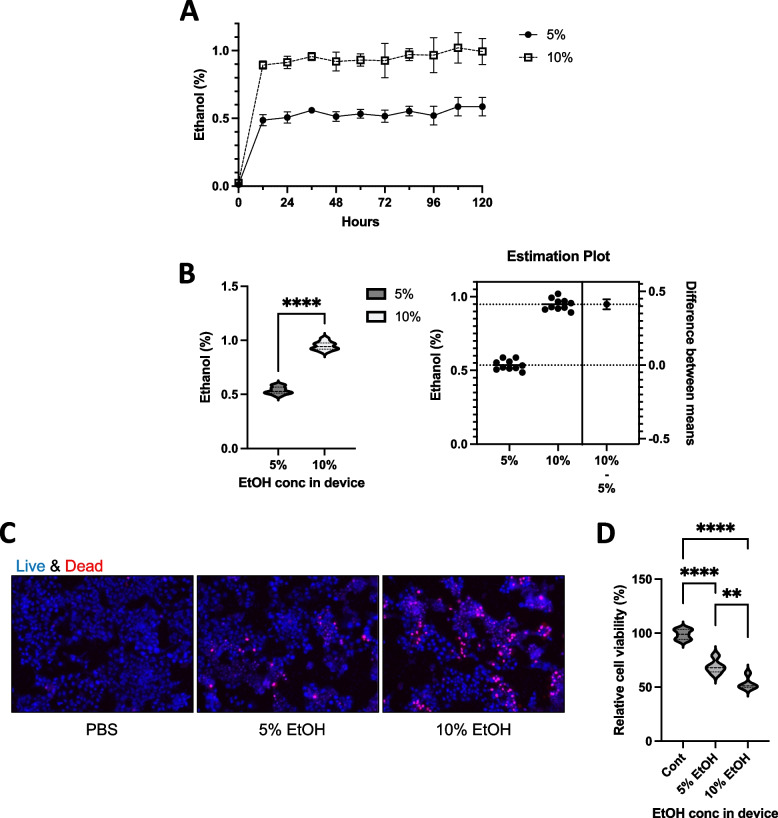
Long-term exposure to ethanol using PDMS devices. **a** 5% or 10% ethanol was added to the storage space of the container. The devices were placed into 6-well plates containing the cell culture medium, and the ethanol concentration in the culture medium was measured at the indicated time points. **b** The average concentration in the culture medium of panel A was calculated (*n* = 30). **c** PDMS devices containing PBS and 5% or 10% ethanol were placed in a L-02 cell-containing culture plate and incubated for 72 h, after which live and dead cells were checked. **d** PDMS devices containing PBS and 5% or 10% ethanol were placed in a culture plate and incubated for another 72 h, after which cell viability was measured (*n* = 5)

We tested the ability of the PDMS device to release ethanol gradually and constantly into a cell culture system. Following the introduction of PDMS devices with 5% or 10% ethanol, the ethanol concentration in the culture media steadily increased until 12 h and then remained constant for 120 h (Fig. 3A). While PDMS devices containing 5% ethanol induced approximately 0.54% ethanol concentration in the culture medium, those with 10% ethanol induced approximately 0.95% ethanol concentration. The ethanol concentration in the PDMS devices considerably influenced that of the culture medium (Fig. 3B). To check the long-term ethanol-releasing function of the PDMS devices, we placed PBS, 5%, or 10% ethanol-containing PDMS devices in a cell-containing culture plate and incubated for 72 h to evaluate cytotoxicity. Long-term exposure to 5% ethanol (approximately 0.54% ethanol concentration in the medium) induced cytotoxicity with approximately 69% cell viability (Fig 3C and D), and exposure to a constant 10% ethanol in PDMS (0.95% ethanol concentration in the medium) resulted in stronger cytotoxicity with approximately 53% cell viability. These results suggest that PDMS devices are suitable for long-term ethanol treatment in cells with a controllable ethanol concentration and could be used as a tool to examine the cellular and molecular mechanisms of chronic ethanol effects in cellular systems.

### Long-term treatment of ethanol alters cytokine levels

The release of proinflammatory cytokines from liver cells is a well-known pathogenesis of alcoholic liver disease [19]. Cytokines trigger considerable changes in the gene expression of their target cells to induce apoptotic damage and produce reactive oxygen species, thereby emphasizing the importance of understanding and controlling alcoholic liver disease.

**Fig. 4:**
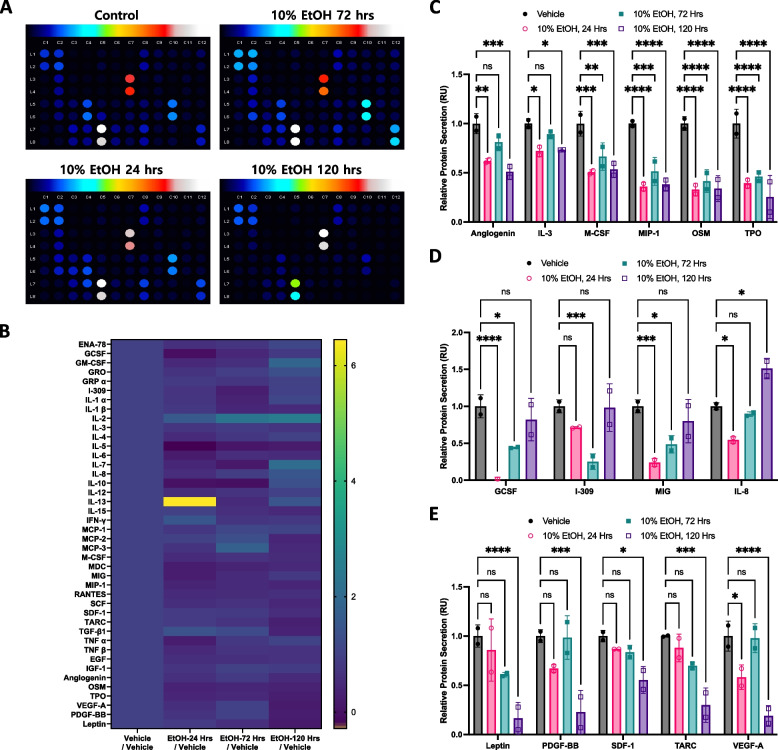
Long-term ethanol exposure alters cytokine levels. **a** PBS or 10% ethanol-containing PDMS devices were placed in cell culture plates and incubated for 24 or 120 h. Then, cytokine levels of L-02 cells were measured from a culture medium. Quantitation of secreted proteins after ethanol treatment was performed by analyzing the membrane-based cytokine array. The density of dots was determined with Protein Analyzer software with Image J. **b** Heat map of the cytokine array showed the relative secretion of the indicated proteins compared to the control. **c** Levels of secreted proteins that showed early changes in protein expression that remained altered over time. One-way ANOVA revealed statistical significance. ns: non-significant, * *p*<0.05, **:*p*<0.01, *** *p*<0.0005; (**d**) Levels of secreted proteins whose secretion was significantly reduced after 24 h of treatment but recovered or even increased at 120 h after ethanol treatment. One-way ANOVA revealed statistical significance. ns: non-significant, * *p*<0.05, **:*p*<0.01, *** *p*<0.0005 (e) Secretion of cytokines that did not change in the initial 24 h of ethanol treatment but decreased over 120 h of exposure. One-way ANOVA revealed statistical significance. ns: non-significant, *: *p*<0.05, **:*p*<0.01, ***: *p*<0.0005

The L-02 normal liver cell line has been utilized for in vitro alcohol stimulation [20, 21]; however, most studies have focused only on short exposure times (<48 h) owing to technical difficulties. We previously reported that the prolonged release of constant alcohol is a more reliable model for the study of in vitro chronic alcohol exposure during mammalian cell cultivation [17]. Because the cytokine release profile after chronic alcohol exposure to L-02 liver cells has also not been reported, we placed 10% ethanol-containing PDMS devices in L-02 cell-containing culture plates for up to 120 h for sustained release of ethanol to a final concentration of approximately 0.95% in the culture medium and measured the secreted cytokine levels (Fig. 4A and B).

Some cytokines showed early changes in protein expression, which remained altered over time (Fig. 4C). For example, angiogenin levels decreased by nearly 40% after 24 h of ethanol release. Angiogenin in hepatocellular carcinoma plays a crucial role in tumor vasculature construction [22]; however, its role in normal liver physiology has been challenging to elucidate. Because angiogenin exhibits enzymatic activity and plays an important role in various signaling pathways related to cell proliferation and survival [23], we hypothesized that normal hepatocyte function would be diminished in chronic ethanol. Secretion of IL-3 and macrophage colony-stimulating factor (M-CSF) from L-02 cells was also notably reduced by ethanol treatment, and the levels remained low for up to 120 h. While IL-3 plays pleiotropic roles in hematopoietic and immune cell differentiation [24], its role in the liver is not well understood. The macrophage colony-stimulating factor is a common-chain cytokine that regulates inflammation. Because M-CSF has been well studied in macrophage regulation, its role in hepatic macrophage control of the liver is clearly elucidated [25]. Macrophage inflammatory protein-1α is a highly expressed protein in transplanted or ischemic injury liver for controlling the immune response [26]. Oncostatin M (OSM) is a key regulator involved in the early development of the liver in fetuses and the growth of hepatocarcinomas in adults [27]. As a major organ-producing hematopoietic hormone, the liver also secretes thrombopoietin (TPO) in response to external signals [28]. Our results indicated that the amount of TPO in L-02 cells was also downregulated in the presence of ethanol.

The secretion of some cytokines was considerably reduced at 24 h, an early stage of ethanol treatment, but recovered or even increased at 120 h after ethanol treatment (Fig. 4D). Granulocyte-colony stimulating factor (G-CSF), a granulopoietic stimulant to mobilize hematopoietic stem cells, is utilized in liver failure [29]. Protein secretion of G-CSF dramatically decreased during the first day of ethanol treatment but recovered 120 h after ethanol release, implying that the decrease in G-CSF secretion is not a long-lasting effect of chronic alcohol exposure in liver cells. I-309 and Mig proteins also showed an early decrease in secretion from L-02 liver cells, but their level recovered 120 h after ethanol treatment, indicating that the amount of cytokines is not significant at the end. I-309, expressed in *CCL1*, plays a role in Treg regulation via FOXp3 upregulation [30]. *CXCL9* encodes the MIG protein, which is an anti-proliferative and anti-migratory cytokine against liver fibrosis in vivo [31]. IL8 is activated in chronic liver disease [32]. We found that IL8 decreased on the first day of ethanol treatment but increased after 120 h.

We also analyzed the secretion of cytokines that did not change in the initial 24 h of ethanol treatment but decreased after 120 h of exposure. The secretion of representative proteins, Leptin, PDGF-BB, SDF-1, and VEGF-A, was not altered within three days; however, their levels considerably decreased after 120 h (Fig. 4E). Leptin is a pleiotropic hormone derived from adipose tissue that is vital for regulating fat deposition in the liver [33]. Platelet-derived growth factor-BB (PDGF-BB) stimulation is associated with liver fibrosis [34]; however, its physiological role in liver function includes the regulation of hepatic stellate cells [35]. Stromal cell-derived factor-1 (SDF-1) promotes the proliferation and invasion of cancer cells but is also expressed in normal hepatocytes for liver homeostasis [36]. Thymus and activation-regulated chemokine (TARC) selectively recruits CCR4-expressing T cells and functions in the pathogenesis of lethal liver damage from bacterial infection [37, 38]. Vascular endothelial growth factor-A (VEGF-A) is known to be a proinflammatory and profibrogenic factor in chronic liver disease [39], and its role in NAFLD has recently received attention in the [40, 41].

Our results indicated changes in the protein expression of representative cytokines secreted by L-02 hepatocytes after chronic alcohol exposure for over 120 h. M-CSF and macrophage inflammatory protein-1α, which are involved in regulating immune responses in the liver, decreased in secretion after the initial 24 h of ethanol treatment and continued until 120 h. In contrast, cytokines such as GCSF and IL8, which decreased in the first 24 h of ethanol treatment, recovered to their initial levels after 120 h. The expression of PDGF-BB and SDF-1, which play important roles in regulating hepatic stellate cell homeostasis, decreased only after 120 h. Therefore, adopting an appropriate method to analyze the chronic response of hepatocytes to ethanol is crucial. This could be achieved with an ethanol-releasing device, as described in the present study.

### Long-term treatment of ethanol changes the physiology of hepatocytes

**Fig. 5:**
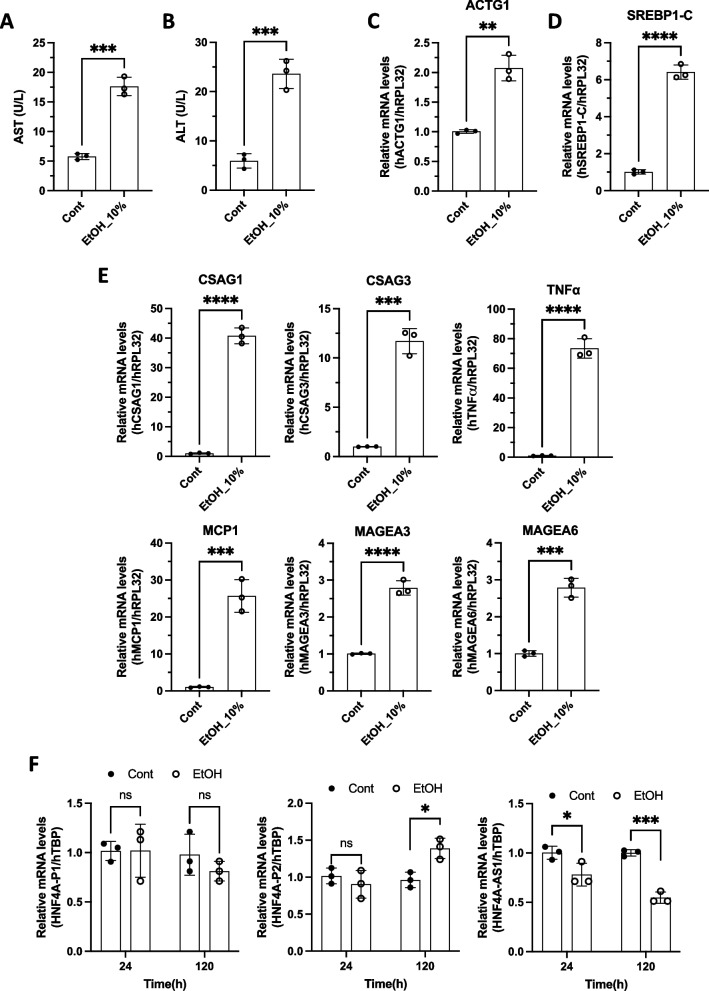
Long-term ethanol exposure changed the gene expression of hepatocytes. **a**-**e** PBS or 10% ethanol-containing PDMS devices were placed in cell culture plates containing L-02 cells and then incubated for 120 h. **a**, **b** AST and ALT levels were measured in the culture medium. **c**-**e** The specified mRNA levels were measured using real-time PCR. (F) PBS or 10% ethanol-containing PDMS devices were placed in L-02 cell-containing cell culture plates, and the indicated RNA levels were measured after 24 or 120 h of incubation

The release of hepatic transaminases is a primary indicator of hepatocellular damage [42]. In this study, ethanol-induced hepatoxicity was assessed using an ethanol-release device by measuring the release of alanine transaminase (ALT) and aspartate aminotransferase (AST) into the culture medium. The levels of ALT and AST increased approximately 3–4-fold in the ethanol-releasing devices compared to those in the non-ethanol-treated control after 120 h of incubation with ethanol using the indicated methods (Fig. 5A and B). We investigated the expression of various genes that were recognized as important indicators for alcohol-related hepatocellular carcinoma to check whether long-term ethanol exposure using PDMS ethanol-releasing device systems exhibits similar cellular physiologies as patients-derived samples show [43, 44]. Actin gamma 1 (ACTG1) mRNA levels, recognized as a biomarker for alcohol-associated hepatocellular carcinoma, were considerably increased by long-term ethanol treatment with PDMS devices for 120 h (Fig. 5C). Recent studies have indicated that ethanol increases fatty acid synthesis in hepatocytes by regulating lipid metabolism-associated transcription factors, such as sterol regulatory element-binding protein 1c (SREBP1c), which promotes fatty acid synthesis via upregulation of lipogenic genes [45]. Long-term ethanol treatment of PDMS devices increased SREBP1c mRNA levels by approximately 6-fold relative to the control (Fig. 5D). The chondrosarcoma-associated gene family (CSAG) is frequently activated in many tumors, including alcohol-related hepatocellular carcinoma (HCC) [44]. The ethanol-releasing device-mediated chronic alcohol treatment dramatically upregulated CSAG1 and CSAG3 expression levels (Fig. 5E). Tumor necrosis factor-alpha (TNFα), a significant factor in the development of alcohol-induced liver injury, and the C motif chemokine ligand 2 (CCL2, also known as MCP1) mediate alcohol-induced inflammatory cell activation [46]. Chronic ethanol treatment increases the expression of TNFα and MCP1 (Fig. 5E). Melanoma-associated antigen A3 (MAGEA3) and MAGEA6 show higher expression levels in HCC tumors than in normal livers [44]. Long-term ethanol exposure induced increased expression of MAGEA3 and MAGEA6 compared to that in the control (Fig. 5E). In human alcoholic hepatitis, hepatocyte nuclear factor 4 alpha (HNF4α) was the most inhibited liver-enriched transcription factor in human alcoholic hepatitis [47]. HNF4 α is responsible for the transcriptional activation of mature hepatocyte-specific genes and plays a role in preserving hepatocellular homeostasis during chronic liver injury [47]. The mRNA levels of HNF4α -P1, an isoform of HNF4α, remained unchanged in alcoholic hepatitis, whereas HNF4α -P2 showed dramatic upregulation in the livers of patients with alcoholic hepatitis. Indeed, the expression of the long noncoding RNA HNF4A-AS1 is downregulated in patients with alcoholic hepatitis [47]. To check if our ethanol-releasing system can also show similar expression patterns of HNF4α in livers from patients with alcoholic hepatitis, we chronically treated the cells with ethanol for 24 or 120 h using our PDMS device. HNF4α -P1 mRNA levels did not change in the presence or absence of alcohol or after treatment for 24 or 120 h (Fig. 5F). HNF4α -P2 was upregulated when ethanol was treated for 120 h. Further, ethanol treatment for 24 or 120 h downregulated HNF4A-AS1 expression levels. These results correlate with HNF4α expression levels in the livers of patients with alcoholic hepatitis.

These results indicate that the PDMS device-mediated long-term ethanol treatment can mimic chronic alcohol-induced hepatocellular physiology.

## Discussion

Alcoholic liver disease is the most frequent cause of alcohol-induced deaths [48], with numerous investigations establishing a firm causal connection between persistent ethanol consumption and human tissue damage [49]. However, unlike experiments conducted in living organisms, lab-based studies on hepatocytes face constraints in consistently subjecting cells to ethanol, given its volatile nature. The current techniques are cumbersome, involving periodic introduction of ethanol or ethanol-saturated air within the incubation chamber.

We aimed to overcome the limitations of the existing treatments by utilizing a simple ethanol carrier design. Our PDMS devices maintain consistent ethanol concentrations for at least three days and can be reused for up to 20 cycles, providing a simple and effective solution to prevent rapid fluctuations in ethanol levels due to evaporation. The PDMS devices in our study were designed for a 6-well plates system. However, their size can be adjusted for high-throughput methods if needed. Further examination of different-sized PDMS devices would be beneficial for delving into the cellular and molecular mechanisms related to diseases associated with chronic alcohol exposure.

Given that the experiment was conducted at a typical cell culture temperature of 37 ℃, it is likely that the rate of ethanol evaporation was significantly increased. This would have accelerated the release of ethanol from the PDMS device. However, at the same time, the higher temperature would also increase the rate of ethanol evaporation from the surface of the culture medium, possibly balancing out the two processes and resulting in the ethanol concentration in the culture medium not increasing as anticipated. This could be an important factor in explaining the observed plateau in ethanol concentration within the cell culture fluid.

We demonstrated that our system effectively replicates actual chronic alcohol consumption-mediated liver conditions in cellular models. However, the analysis of annotated or putative alcohol-related HCC biomarkers in L-02 and HepG2 cells after chronic alcohol exposure using PDMS devices revealed that not all annotated or key biomarkers may correlate with our cellular system. Given the diverse cell types in the liver, including hepatocytes, hepatic stellate cells, Kupffer cells, and liver sinusoidal endothelial cells, further analyses with diverse liver cells are necessary to mimic the actual chronic alcohol consumption scenario and determine the exact cellular and molecular mechanisms.

Our ethanol-releasing PDMS device will help address the cellular and molecular mechanisms of chronic alcohol-mediated liver diseases. And our work will stimulate research into the development of various devices and methods required to reproduce phenomena at the organismal and cellular levels.

## Methods

### Cell culture and ethanol treatment

We cultured HepG2 cells (Korean Cell Line Bank) in Eagle’s minimal essential medium (Sigma-Aldrich) supplemented with 10% fetal bovine serum (FBS, Sigma-Aldrich) and 1% antibiotics (Sigma-Aldrich). We cultured L-02 cells in Dulbecco’s modified Eagle’s medium (Sigma-Aldrich) supplemented with 10% FBS and 1% antibiotics. Cells were maintained in a humidified incubator with 95% ambient air and 5% CO_2_ at 37 ℃.

For ethanol treatment using direct addition, we seeded the cells in 96-well plates or 12-well plates and incubated them for 24 h. Subsequently, the specified ethanol concentration (Sigma-Aldrich, St. Louis, MO, USA) was directly added to the culture medium, and the mixture was incubated for the designated time. For the PDMS device system, the specified ethanol concentration was added to PDMS devices, which were then placed in 6-well culture plates. Ethanol was added to the PDMS device every 48 h.

### Cell viability and ethanol measurement

Cell viability was assessed using a Cell Counting Kit-8 (Dojindo, Kumamoto, Japan) following the manufacturer’s instructions. Ethanol-containing phosphate-buffered saline (PBS) or culture medium was obtained at specified time points, and the ethanol concentration was determined using an Ethanol Assay Kit (BioAssay Systems, Hayward, CA, USA) with an Infinite M nano microplate reader (Tecan, Zurich, Switzerland) according to the manufacturer’s instructions.

### Cytokine analysis

The ethanol-containing PDMS devices were placed in L-02 cell culture plates for specified durations. Following the manufacturer's instructions, a cytokine array was performed after clarifying the supernatant at 15, 000 × g for 30 min (AAH-CYT-3; RayBiotech Life, Peach Tree Corners, GA, USA). The ImageJ software (NIH, Bethesda, MD, USA) was used to quantify arbitrary protein quantities.

### RNA quantification

Total RNA was extracted from cells using TRIzol^TM^ reagent (Invitrogen^TM^). RNA was reverse-transcribed using GoScript^TM^ Reverse Transcriptase (Promega) with oligo(dT) primers, following the manufacturer’s instructions. mRNA levels were detected by quantitative real-time PCR using a QuantStudio 3 real-time PCR instrument (Applied Biosystems) and SYBR Green PCR Master Mix (Thermo Fisher Scientific).

### Statistical analysis

Statistical parameters, including definitions and exact values of n (number of biological repeats), distributions, and deviations, are reported in the figures and their corresponding legends. All quantitative data are presented as mean ± standard error of the mean. Comparisons between the two groups were conducted using two-tailed unpaired Student’s *t*-tests. For comparisons between more than two groups, one-way analysis of variance was used with Tukey’s post-hoc test. Two-way analysis of variance was used with Tukey’s post-hoc test to estimate the mean differences between groups that had been split into two independent variables. Statistical significance was set at *p* < 0.05. Statistical analyses were performed using GraphPad Prism software.

## Data Availability

No datasets were generated or analysed during the current study.

## Abbreviations

PDMS: Polydimethylsiloxane
PLA: Polylactic acid
M-CSF: Macrophage colony-stimulating factor
OSM: Oncostatin M
TPO: secretes thrombopoietin
G-CSF: Granulocyte-colony stimulating factor
PDGF-BB: Platelet-derived growth factor-BB
SDF-1: Stromal cell-derived factor-1
ALT: Alanine transaminase
AST: Aspartate aminotransferase
ACTG1: Actin gamma 1
SREBP1c: Sterol regulatory element-binding protein 1c
MAGEA3: Melanoma-associated antigen A3
HNF4α: hepatocyte nuclear factor 4 alpha
